# Variant Prevertebral Muscle: Unique Cadaveric Findings

**DOI:** 10.7759/cureus.1515

**Published:** 2017-07-25

**Authors:** Jocelyn R Gonzales, Joe Iwanaga, Rod J Oskouian, R. Shane Tubbs

**Affiliations:** 1 Neurosurgery, Seattle Science Foundation; 2 Seattle Science Foundation; 3 Neurosurgery, Complex Spine, Swedish Neuroscience Institute

**Keywords:** back, muscles, anatomy, embryology, neck, atlas, physical therapy, tendon, cervical, occipital cervical junction

## Abstract

The levator scapulae muscle typically runs from the transverse process of the atlas to the superior angle of the scapula. In this paper, we describe a rare finding identified during a dissection of a male cadaver, wherein a continuation of the right levator scapulae ran past its normal attachment to the C1 transverse process, fusing with the inferior attachment of the rectus capitis lateralis muscle. No variants were found on the opposite side, and the innervation of the levator scapulae muscle variant was typical of that of a normal levator scapulae. We also describe other related variants of the cranial levator scapulae muscle, hypothesize the embryologic origin of our finding, and finally discuss potential clinical relevance of levator scapulae muscle variants.

## Introduction

In most instances, the rectus capitis lateralis muscle runs superiorly from the transverse process of the atlas and inserts onto the jugular process of the occipital bone. The levator scapulae muscle originates from the transverse processes of the upper four cervical vertebrae and inserts onto the superior angle of the scapula [1]. In this paper, we describe a previously unreported muscle variant of the levator scapulae muscle. PubMed literature reviews of variants of the levator scapulae or rectus capitis lateralis have reinforced our belief that this is the first reported case of its kind. We will additionally describe relevant literature regarding other known cranial variants of the levator scapulae muscle, potential embryologic origins, and its clinical relevance.

## Case presentation

**Figure 1:**
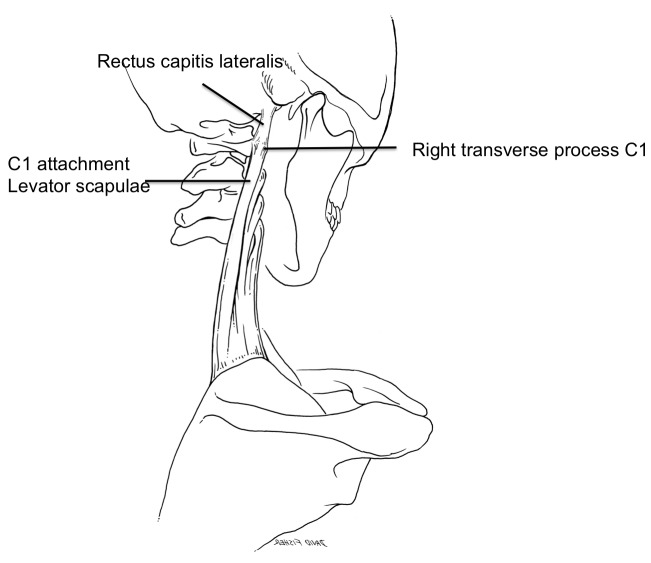
Schematic drawing of the case reported herein. Note the continuation of the upper fibers of the levator scapulae muscle continuing on past the transverse process of C1 to fuse with the inferior fibers of the rectus capitis lateralis muscle.

**Figure 2:**
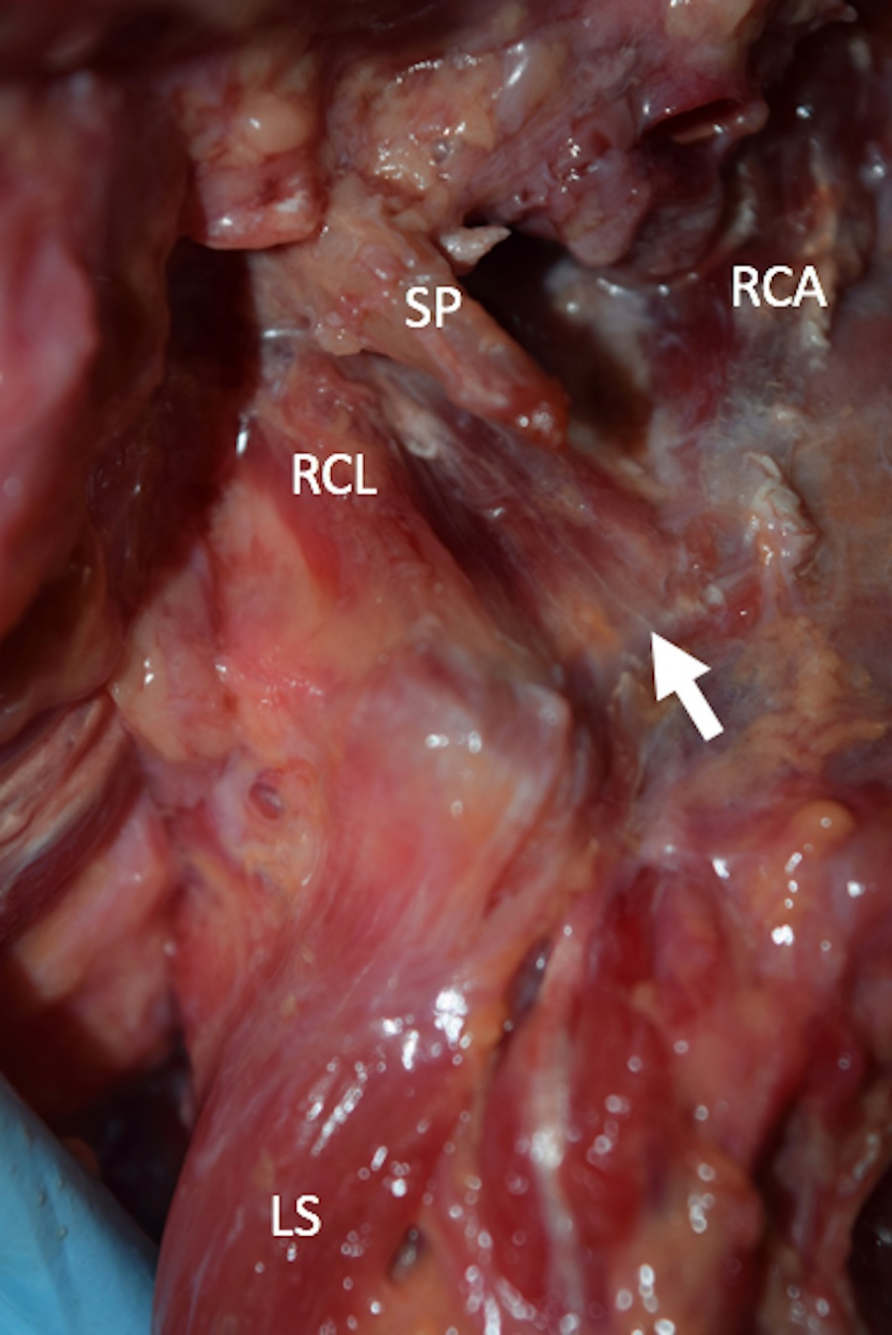
Anterolateral view of the muscle variant (arrow). SP: styloid process, RCA: rectus capitis anterior, RCL: rectus capitis lateralis, LS: levator scapula.

**Figure 3:**
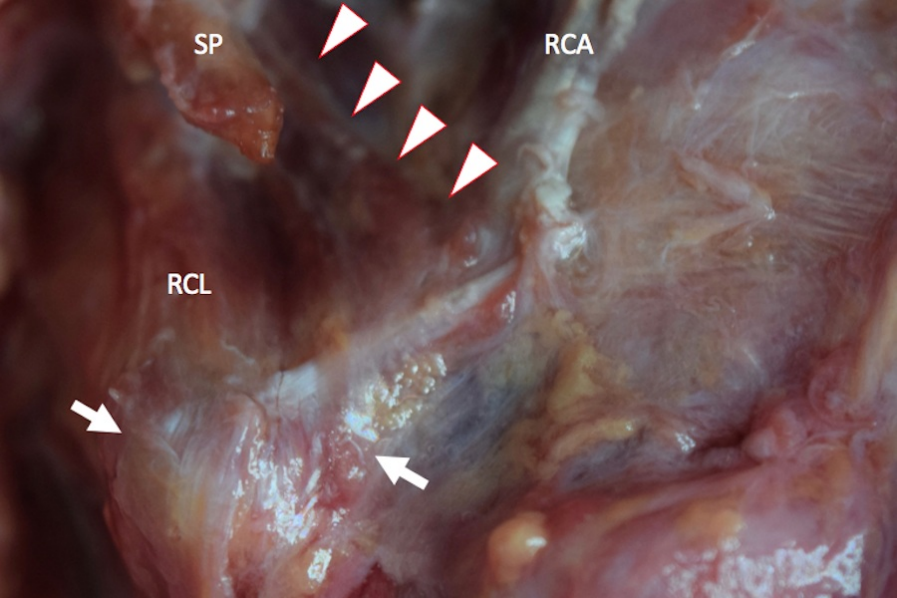
Magnified picture of figure 1. Note some of the fibers of the RCL and muscle variant continued to the levator scapula (arrows). SP: styloid process, RCA: rectus capitis anterior, RCL: rectus capitis lateralis.

A 77-year-old-at-death, fresh, frozen, Caucasian male cadaver was dissected. During dissection of the right prevertebral region, a muscle variant was discovered. This anatomical anomaly was seen as a continuation of the levator scapulae muscle’s upper fibers attaching onto the transverse process of C1, continuing on to fuse with the inferior attachment of the rectus capitis lateralis muscle (Figure 1). The rectus capitis lateralis muscle on the right side arose from the anterior surface of the transverse process of the atlas and inserted into the jugular process of the occipital bone. The rectus capitis anterior muscle on the right side arose from the anterior surface of the lateral mass of the atlas and passing superior-medially, inserted into the inferior surface of the basilar part of the occipital bone just in front of the foramen magnum. The muscle variant of the levator scapulae had fibers found between the origins of these two muscles, which originated from the anterior surface of the transverse process medial to the origin of the rectus capitis lateralis muscle, and passing superolaterally, inserted onto the jugular process of the occipital bone medial to the insertion of the rectus capitis lateralis muscle (Figures 2-3). There were no muscle variations of the levator scapulae on the left side or other prevertebral variants on this side. The variant muscle was not found to have a separate nerve innervation from that of the levator scapulae muscle.

## Discussion

While no previous studies on muscle variants of the rectus capitis lateralis were found in our review, there are several case reports regarding muscle variants of the levator scapulae. For proximal variations specifically, one cadaveric study found that the left levator scapulae of an 88-year-old female extended to the left mastoid process, just posterior to the mastoid process attachment of the sternocleidomastoid. The authors of this case speculated a potential relationship between this muscle variant and various neck and shoulder pain syndromes [2]. Another case report found in a 71-year-old-at-death female was found to have a variant of the left levator scapulae wherein the muscle gave rise to an accessory head that inserted via a flat aponeurotic band into the ligamentum nuchae, the tendon of the rhomboideus major, and the superior aspect of the serratus posterior superior muscle [3]. While the authors acknowledge a lack of information regarding the clinical relevance of such a variation of the levator scapulae muscle, a similar speculation that the variant could give rise to neck and shoulder pain was made. Other variant proximal attachments have included the occipital bone [4].

Embryologic origin

The embryonic origins of muscle cells which form the skeletal muscles have been well studied. However, debate over how much of the dermomyotome becomes the myotome, the precursor to the epaxial muscles, has occurred. The most recent studies have found that the myotome arises exclusively from the dorsomedial and ventromedial lips of the dermomyotome or that all four margins of the dermomyotome contribute to the formation of the myotome. As these studies largely concern rodent and avian anatomy, whether these claims hold true for humans is unclear [5]. Eventually, these muscles form into unique back and neck muscles [6]. Interestingly, the study by Venters, et al. has shown that the myotome develops asymmetrically; the ventral region maturing at a faster rate than the dorsal region [7].

A study by Mekonen, et al. used 3D reconstruction and remodeling to analyze the growth of each specific epaxial muscle in embryos. By using general and immunohistochemical staining, they followed the differentiation of the dorsomedial lip into the unique epaxial muscles. This study determined the development of the medial cervical muscles to occur in the eighth week, specializing into rod-like ventrolateral and spade-like dorsomedial portions, and additionally found that all three columns in the cervical region followed the same trend of development and specialization [8].

Considering the consistency of the development of medial cervical muscles as found by Mekonen, et al., we speculate that the variant muscle reported herein originated in a similar way and due to its juxtaposition to the rectus capitis lateralis muscle would have a similar embryology.

Clinical relevance

Behsrin and Maguire detailed the physiological involvement of the levator scapulae muscle in shoulder movement. From electromyography, the levator scapulae muscle has been found to contract concentrically during the first 90 degrees of shoulder abduction and contract eccentrically during the last 90 degrees. They speculated that the substantial involvement of the levator scapulae muscle in shoulder abduction could mean that cervical pain due to shoulder abduction might be due to variants of the levator scapulae [9].

Cervical dystonia (CD) is another condition that can involve the levator scapulae muscle and surrounding muscle groups. CD is characterized by involuntary contraction that results in the head being rotated to either side. Polymyographic recordings of patients with this condition revealed ipsilateral levator scapulae muscle involvement in 30% of cases. Erro, et al. speculated that variants of the levator scapulae muscle might contribute to or exacerbate CD [10]. Additional attachments of the levator scapulae muscle into the rectus capitis lateralis muscle as found in the case presented herein might result in greater flexion of the craniocervical junction.

## Conclusions

In this article we described, to our knowledge, the first case description of the levator scapulae muscle attaching onto the inferior aspect of the rectus capitis lateralis muscle. Additionally, we described other muscle variants of the levator scapulae, specifically, its cranial attachments, the potential embryologic origin of the muscle variant, and its clinical relevance.
